# Primary oxidative phosphorylation defects lead to perturbations in the human B cell repertoire

**DOI:** 10.3389/fimmu.2023.1142634

**Published:** 2023-07-07

**Authors:** Eliza M. Gordon-Lipkin, Payal Banerjee, Jose Luis Marin Franco, Tatiana Tarasenko, Shannon Kruk, Elizabeth Thompson, Derek E. Gildea, Suiyuan Zhang, Tyra G. Wolfsberg, NISC Comparative Sequencing Program, Willy A. Flegel, Peter J. McGuire

**Affiliations:** ^1^ Metabolism, Infection and Immunity Section, National Human Genome Research Institute, National Institutes of Health, Bethesda, MD, United States; ^2^ Bioinformatics and Scientific Programming Core, National Human Genome Research Institute, National Institutes of Health, Bethesda, MD, United States; ^3^ NIH Intramural Sequencing Center, National Human Genome Research Institute, National Institutes of Health, Bethesda, MD, United States; ^4^ Department of Transfusion Medicine, NIH Clinical Center, National Institutes of Health, Bethesda, MD, United States

**Keywords:** B cells, mitochondria, viral infection, VirScan, RNAseq analysis

## Abstract

**Introduction:**

The majority of studies on oxidative phosphorylation in immune cells have been performed in mouse models, necessitating human translation. To understand the impact of oxidative phosphorylation (OXPHOS) deficiency on human immunity, we studied children with primary mitochondrial disease (MtD).

**Methods:**

scRNAseq analysis of peripheral blood mononuclear cells was performed on matched children with MtD (N = 4) and controls (N = 4). To define B cell function we performed phage display immunoprecipitation sequencing on a cohort of children with MtD (N = 19) and controls (N = 16).

**Results:**

Via scRNAseq, we found marked reductions in select populations involved in the humoral immune response, especially antigen presenting cells, B cell and plasma populations, with sparing of T cell populations. *MTRNR2L8*, a marker of bioenergetic stress, was significantly elevated in populations that were most depleted. *mir4485*, a miRNA contained in the intron of *MTRNR2L8*, was co-expressed. Knockdown studies of *mir4485* demonstrated its role in promoting survival by modulating apoptosis. To determine the functional consequences of our findings on humoral immunity, we studied the antiviral antibody repertoire in children with MtD and controls using phage display and immunoprecipitation sequencing. Despite similar viral exposomes, MtD displayed antiviral antibodies with less robust fold changes and limited polyclonality.

**Discussion:**

Overall, we show that children with MtD display perturbations in the B cell repertoire which may impact humoral immunity and the ability to clear viral infections.

## 1 Introduction

Immunometabolism as a discipline encompasses the interplay between metabolism and immunocyte proliferation, differentiation, and function (1). Over the past decade, the field has delivered significant advances, with mitochondria taking center stage. To date, the majority of immunometabolism studies have been conducted in mouse models, emphasizing the need for translational immunometabolism. Moreover, the role of oxidative phosphorylation (OXPHOS) in human immune cell populations, specifically B cells, is nearly absent from the biomedical literature.

Since one of the main goals of biomedical research is to improve the health and well-being of humankind, all biomedical research utilizing model organisms and systems is directed at human translation. Despite the widespread availability of various models, the human system displays its own extensive assortment of phenotypes due to deleterious variants in genes, and thus represents the ultimate experimental model system (2). Indeed, the study of rare diseases has greatly contributed to our understanding of biologic mechanisms and remains a viable model for defining complex disease pathophysiology (3).

Primary mitochondrial dysfunction, known as mitochondrial disease (MtD), is a group of disorders caused by deleterious variants in nDNA or mtDNA genes involved in OXPHOS, the main pathway for energy generation in cells (4). Due to the ubiquity of the mitochondria, the clinical phenotype of MtD is pleiotropic, involving organs and systems with considerable energy needs. The immune phenotype of patients with MtD is emerging and manifests as susceptibility to infection and immune dysfunction (5), making this population ideal for translational immunometabolism investigations.

To define the role of OXPHOS in human immune cells, we began by performing single cell RNAseq in peripheral blood mononuclear cells from children with MtD. Cluster analysis and differential gene expression revealed markers of cellular bioenergetic stress in select immune cell populations with depletion. Based on our findings, we hypothesized that defects in OXPHOS would manifest as perturbations in the humoral immune response. To answer this question, we profiled the antiviral antibody repertoire *via* phage display immunoprecipitation sequencing (i.e., VirScan) in children with MtD due to their deleterious relationship with viral infections (6).

## 2 Methods

### 2.1 Subjects

All participants were consented and enrolled in an IRB approved longitudinal natural history study of viral infection and immunity in children with MtD (NIH MINI Study, NCT01780168, www.clinicaltrials.gov) and evaluated at the National Institutes of Health Clinical Center. MtD diagnosis was made by referring physicians and included clinical testing and identification of deleterious variants. The characteristics of the cohort are shown in **Table S1**. The mean age of the control and MtD cohorts were 6.4 and 6.6 years of age, respectively, with similar proportions of male and female subjects. MtD diagnoses included: 11 Leigh syndrome, 4 Leigh-Like syndrome, 1 Kearns-Sayre syndrome, and 4 MtD not otherwise specified (NOS). Molecular diagnoses and CNS lesions, a potential deleterious consequence of viral infection (7), are also indicated. Three patients with MtD were being treated with immunoglobulin replacement therapy (IRT). Up to 20% of individuals with MtD receive IRT for a clinical diagnosis of immunodeficiency or a history of repeated infections (8). Since the antiviral antibody profiles of these patients represent the collective exposures of the donors, these samples were analyzed separately.

### 2.2 OCR and ECAR measurement

Oxygen consumption rate (OCR) and extracellular acidification rate (ECAR) were measured using a Seahorse XF96 analyzer (Seahorse Bioscience). Lymphoblastoid cell lines were attached with Cell-Tak (Corning) according to manufacturer’s instructions at concentration 0.5 million cells/well in Seahorse BASE media with proprietary additives. Oxygen consumption rate (OCR) and extracellular acidification rate (ECAR) were determined using the Mitostress kit (Seahorse Biosciences) according to the manufacturer’s standard protocol. OCR and ECAR were calculated and recorded by the Seahorse XF-96 software.

### 2.3 scRNAseq of peripheral blood mononuclear cells

scRNA-Seq libraries were prepared from 16k cells using a 10X Genomics Chromium device and Chromium Single Cell 3’ Reagent Kit v3.1 according to manufacturer’s protocol. Libraries were pooled and sequenced on a NovaSeq 6000 DNA sequencer (Illumina, Inc.) using V1.5 chemistry, generating >270M reads per sample. The raw data were processed using RTA 3.4.4. Raw fastq files from single cell sequencing were processed using the CellRanger (10X Genomics Cell Ranger 7.0.1) pipeline. The reads were aligned to the CellRanger_GRCh38_2020-A (https://support.10xgenomics.com/single-cell-gene-expression/software/release-notes/build#GRCh38_2020A) human reference genome. The filtered feature-barcode matrices produced from the 10X pipeline were used for further downstream clustering and analysis. Unsupervised cell clustering was performed by Seurat (4.1.1) (9) in R. (4.1.0). For each sample, the filtered feature-barcode matrix produced from the CellRanger pipeline was read and converted to Seurat object using “Read10X” and “CreateSeuratObject” functions, respectively. Criteria were used to identify gel bead-in-emulsions (GEMs) likely containing mRNAs derived only from a single cell, where GEMs were retained if more than 200 genes and less than 2500 genes were detected. Genes were retained for downstream analysis if they were detected in minimum of three cells. For each sample, the ‘‘NormalizeData’’ was performed to normalize for gene expression followed by ‘‘FindVariableGene’’ function to identify a subset of genes, in this case limited to 2000 genes, that exhibit high cell-to-cell variation. The 8 samples in controland experiment groups were integrated using the ‘‘FindIntergrationAnchors’’ and ‘‘IntegrateData’’ functions with the dimension parameter set to 20. Next, the integrated dataset was scaled, and PCA was performed on this dataset. The first 20 principal components (PCs) were used to perform UMAP to place similar cells together in low-dimensional space. Then the shared nearest-neighbor graph (SNN) was constructed using the “FindNeighbors” function, using the first 20 PCs. The “FindClusters” function, which implements a graph-based Louvain algorithm was applied to identify 11 distinct clusters. The resolution was set to 0.2 for this dataset. The clusters were annotated with the top markers using “FindAllMarkers” function. Classic cell markers were selected as reference after extensive literature and database search. (example - Human Protein Atlas (10) and PanglaoDB (11)). The following genes were used as markers for cell type identity - MS4A1(B cells), CD8B, CD8A (T cells), LYZ (Dendritic cells), GNLY (NK cells), IL7R, BCL11B, MAL (T memory cells/T cells), SLC25A37 (Erythroid-like and erythroid precursor cells), GZMB, PTGDS, PPP1R14B (Plasmacytoid dendritic cells), HBB, HBA1, HBA2 (Red blood cells) and PPBP (Platelets). The differentially expressed genes between the cells from control and the children with MtD were calculated by “FindMarkers”.

### 2.4 LCL miRNA knockdown

LCL cells were transfected using the mirVana miRNA inhibitor protocol (Thermo Fisher, Waltham, MA). mirVana miRNA mimetics are chemically modified double-stranded RNA molecules designed to mimic endogenous microRNAs (miRNAs), resulting in downregulation of translation. LCL cells were seeded at a density of 1x10^6^ cells per well in a 24-well cell culture plate 48 h prior in glucose-free RPMI 1640 (10% fetal calf serum, 40 mM Galactose). At the time of transfection, 30 pmol of mirVana™ miRNA inhibitor 4485-5p was diluted in Opti-MEM^®^ reduced serum medium (final volume: 150 μL). In a separate tube, 9 μL of Lipofectamine™ RNAiMAX Transfection Reagent was diluted in Opti-MEM^®^ Reduced Serum Medium (final volume: 150 μL) and incubated for 5 minutes at room temperature. Combined miRNA inhibitor and Lipofectamine™ RNAiMAX Transfection Reagent (transfection complexes) were incubated for 20 minutes at room temperature. Culture media was removed from the cells and washed once with prewarmed Opti-MEM^®^ reduced serum medium (without antibiotics). The transfection complexes were added dropwise to the cells. The plate was gently shaken to ensure uniform distribution of the complexes. Cells with transfection complexes were incubated at 37°C in a 5% CO_2_ incubator for 48 hours. After the incubation period, the transfection medium was replaced with fresh complete culture medium, RPMI with 10% fetal bovine serum, and the corresponding analyzes were carried out on the transfected cells.

### 2.5 Sample collection for RNA and microRNA extraction and qPCR

LCL cells were harvested at the desired time by centrifugation at 1250 RPM for 5 minutes.

The cell pellet was collected, and RNA extraction was performed. Total RNA, including small RNA molecules, was extracted from the cell pellets using the miRNeasy Micro kit (Qiagen) according to the manufacturer’s instructions. RNA concentration and quality were assessed using a synergy/HTX spectrophotometer and cDNA synthesis proceeded. Reverse transcription was performed for the miRNA4485-5p and the MTRNR2L8 gene using the TaqMan MircoRNA reverse transcription kit. (Applied Biosystems) following the manufacturer’s instructions. Real-time PCR was performed using a CFX96 Touch Real-Time PCR (Bio-Rad). For the miRNA4485-5p amplification reaction, a taqMan microRNA assay from (Applied Biosystems) was used, and for the amplification of the MTRNR2L8 gene, a taqMan RNA assay from (Applied Biosystems) was used. Each reaction was performed in triplicate to ensure reproducibility. Appropriate negative controls (no template and no reverse transcriptase controls) were included to check for contamination and primer-dimer formation. Expression levels of miRNA4485-5p and the MTRNR2L8 gene were normalized to an endogenous control gene of the actin gene ACTB1 using the ΔΔCt method. Data analysis was performed using Maestro analysis software (Bio-Rad) to calculate the change in gene expression.

### 2.6 Apoptosis-Annexin V-FITC staining

LCL cells were harvested at the desired time and counted using a hemocytometer. Cell concentration was adjusted to 0.25x10^6^ cells/mL using complete culture medium. Cell suspension (1000 μL) was transferred to a sterile flow cytometry tube. The tube was spun at 1250 RPM for 5 minutes to pellet the cells. The supernatant was carefully aspirated without disturbing the cell pellet. The cell pellet was resuspended in 200 μL of 1X Annexin V binding buffer and mixed gently. Annexin V-FITC solution (5 μL) was added to the cell suspension, mixed gently, and the cells were incubated in the dark at room temperature for 20 minutes. After the incubation period, 1000 μL of 1X Annexin V binding buffer was added to the cell suspension and mixed gently. Stained cells were analyzed using a CytoFLEX S flow cytometer equipped with appropriate excitation and emission filters for FITC (e.g., 488 nm excitation and 525 nm emission). Appropriate compensation controls were performed using unstained cells and single stain controls to ensure accurate analysis of Annexin V-FITC staining.

### 2.7 Genomic HLA typing

Low to intermediate resolution HLA typing was performed for HLA class II loci (HLA-DRB1, -DQB1, -DRB3, -DRB4, -DRB5) with sequence specific oligonucleotide (SSO) probes (LABType SSO; One Lambda, Canoga Park, CA) (12). The fluorescent intensity of phycoerythrin on each microsphere (applying the Luminex bead technology; Luminex, Austin, TX) was measured with a flow cytometer (LABScan3D, Luminex FLEXMAP 3D; One Lambda). The reaction patterns of the beads were automatically interpreted (Fusion 4.3 IVD software; One Lambda) to identify the antigen assignments by locus for each sample. This HLA typing method was FDA-approved for patient care purposes.

### 2.8 Phage display immunoprecipitation sequencing (VirScan)

Serum was collected, separated, and stored at -80°C until use. Clinical testing was performed by the Department of Laboratory Medicine at the NIH Clinical Center (Bethesda, MD) or Mayo Clinic Laboratories (Rochester, MN). VirScan services were provided by CDI Labs (Baltimore, MtD). In brief, library cloning, sample screening, PCR, and peptide read count data curation are performed as described in Mohan, D., et al. (13). The VirScan library comprises over 110,000 56-mer overlapping peptides encapsulating the human viral proteome. Briefly, screens are performed in 1 mL PBS, pH 7.4 containing phage library (~10^11^ pfu) and 0.2 μL serum (~2 μg IgG). A set of 8 independent buffer alone samples in which serum is not added is included as negative control (beads only). The mixture is rotated overnight at 4°C. The next day, 40 μL of a 1:1 Protein A/G coated magnetic bead slurry is added and rotated for an additional 4 hours at 4°C. The beads are then washed three times with TBS, pH 7.4 containing 0.1% NP-40, and resuspended in 20 μL of a Herculase II Fusion Polymerase PCR1 master mix (Agilent Genomics, Santa Clara, CA). After 20 cycles of PCR, sample-specific barcoding and the Illumina P5/P7 (Illumina, San Diego, CA) adapters are then incorporated during a subsequent PCR2 reaction. PCR2 amplicons are pooled and sequenced using an Illumina NextSeq to obtain single-end 50 nucleotide reads. Demultiplexed reads are aligned to the human peptide library using exact matching. The R software package edgeR (www.r-project.org) then compares the reads in each sample against the buffer alone (beads only) “mock” immunoprecipitations using a negative binomial model. The software returns both a test statistic and fold-change value for each peptide. Enriched peptides (hits) require counts, p-values, and fold changes of at least 15, 0.001, and 5, respectively.

### 2.9 Antiviral antibody response deconvolution algorithm

To account for disproportionate representation of viruses in the library or for antibody cross-reactivity among sequences shared by related viruses, viral exposure was determined by the AntiViral Antibody Response Deconvolution Algorithm (AVARDA) (14). Briefly, AVARDA provides a probabilistic calculation of infection by alignment of all library peptides to each other and to all human viruses.

### 2.10 Statistics

Statistical analyses were performed using R (www.r-project.org), GraphPad Prism (GraphPad Software, San Diego, CA), and Microsoft Excel (Microsoft, Redmond, WA). Online calculators were used for Fisher’s exact test (www.socscistatistics.com/tests/fisher) and Chi square contingency test (5x5 table, www.socscistatistics.com/tests/chisquare2, and unlimited table, www.icalcu.com/stat/chisqtest.html). Data are presented as counts, fold changes, medians, and means ± standard error or standard deviation. Significance was evaluated with the appropriate nonparametric statistical test and is indicated in the figure legends. Multiple testing correction and *post hoc* testing were also performed as indicated in the text and figures. All tests were two-sided and p < 0.05 was considered statistically significant. De-identified data is available upon request from the corresponding author.

## 3 Results

### 3.1 Identification of PBMC subsets in children with MtD by scRNAseq

**Figure 1:**
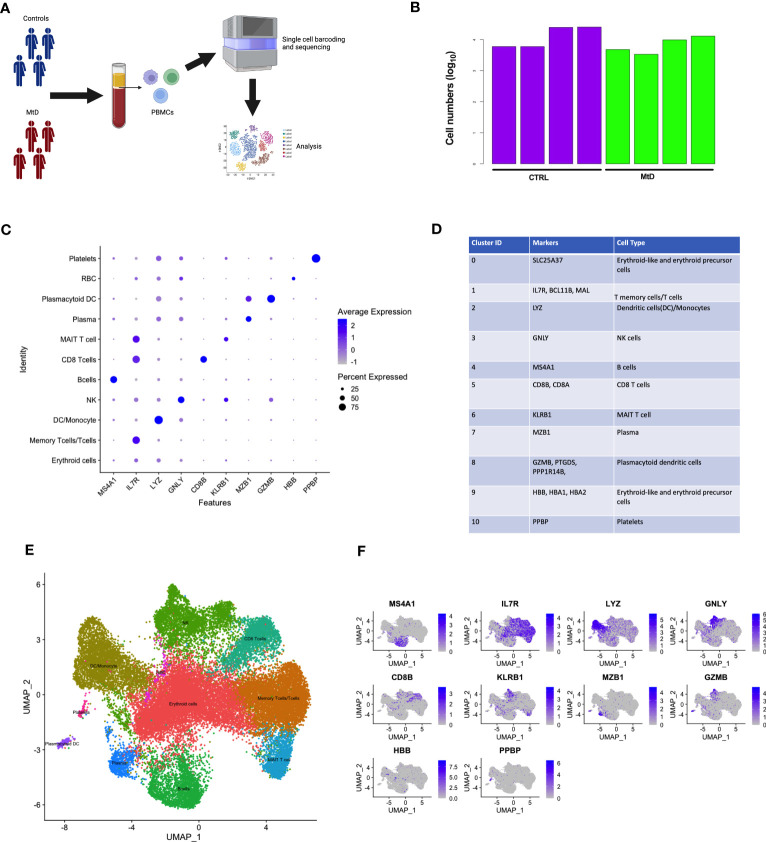
Identification of scRNAseq cell clusters in children with MtD and controls. **(A)** Peripheral blood mono nuclear cells were collected from children with MtD (N = 4) and matched controls (N = 4). Single cells were sorted, followed by isolation of RNA and sequencing. **(B)** Log_10_ cell numbers for analyses from children with MtD and controls. **(C)** Differential gene expression analyses identified cell specific markers. A dot plot for cellular marks and their alignment with various cell populations was constructed. **(D)** Table of RNA markers of various immune cell subsets. **(E)** UMAP clustering of immune cell populations as identified using selected marker genes. **(F)** Feature plots showing the distribution of cellular markers for the identification of immune cell populations.

MtD is marked by pleiotropy, affecting organs and tissues with large energy requirements (15). One major question under investigation in mitochondrial medicine is whether this pleiotropy extends to the immune system. To determine whether primary oxidative phosphorylation deficiency manifested as transcriptional perturbations in immune cell populations, we performed scRNAseq studies in peripheral blood mononuclear cells (PBMCs) from 4 patients with MtD at baseline (Subjects 1,9,14,20, Mean age = 6.5 y/o) (**Table S1**), and healthy controls (N = 4, Mean age = 7.25 y/o) (**Figure 1A**). After filtering for low quality cells, we obtained transcriptomes from 31,584 cells with an average of 11,600 cells for each participant (**Figure 1B**). Viability was > 96% for the samples (**Figure S1A**). To uncover immune cell populations, we performed unsupervised clustering using a shared nearest neighbor approach. *Via* this method, we segregated the cells into 11 major populations (**Figure S1B**). Using established markers (**Figures 1C, D**) (16, 17), we were able to identify the following immune cell populations: erythroid cells (*SLC25A37*), memory T cells/T cells (*IL7R, BCL11B, MAL*), dendritic cells/monocytes (*LYZ*), natural killer cells (*GNLY*), B cells (*MS4A1*), CD8 T cells (*CD8A, CD8B*), mucosal associated invariant T cells (MAIT, *KLRB1*), plasma cells (*MZB1*), plasmacytoid dendritic cells (*GZMB, PTGDS, PPP1R14B*), erythroid-like/erythroid precursor cells (*HBB, HBA1, HBA2*), and platelets (*PPBP*) (**Figure 1E**). To confirm identification of our 11 populations and understand the distribution, we overlayed the expression of these cell population-defining genes back onto our clusters *via* feature plots (**Figure 1F**). As expected, while some markers were more generalized (e.g., IL-7R), others localized to specific clusters (e.g., MS4A1), permitting identification. Overall, clusters were identified by either a single characteristic marker or a combination of multiple markers.

### 3.2 Heterogeneous expression of OXPHOS subunits involved in MtD in immune cells

**Figure 2:**
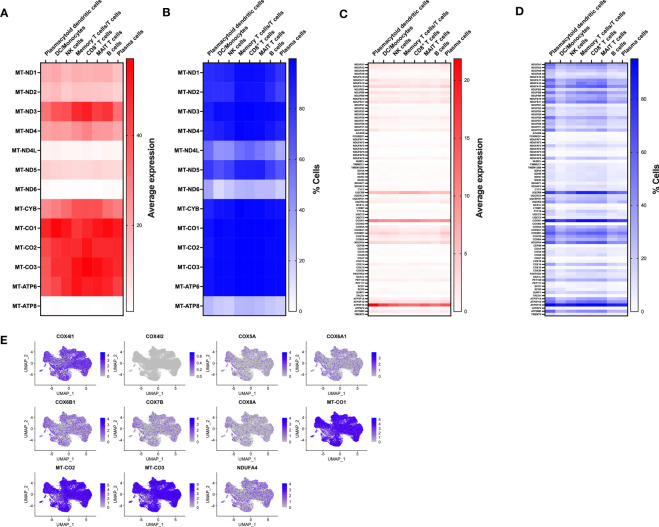
Expression of nDNA and mtDNA genes involved in OXPHOS. **(A)** Transcriptomes from single cell populations were analyzed for the average expression for mtDNA encoded OXPHOS subunits. **(B)** Transcriptomes from single cell populations were analyzed for the % of cells expressing mtDNA encoded OXPHOS subunits. **(C)** Transcriptomes from single cell populations were analyzed for the average expression for nDNA encoded OXPHOS subunits. **(D)** Transcriptomes from single cell populations were analyzed for the % of cells expressing nDNA encoded OXPHOS subunits. **(E)** Feature plot showing the level and distribution of expression of select genes for cytochrome c oxidase (COX, Complex IV).

To gain insight into immune populations that may be affected in MtD, we examined the expression of a select set of nuclear and mitochondrial genes that were either OXPHOS subunits or assembly factors. Deleterious variants in this select set produce MtD. Due to multiple copies of the mitochondrial genome per cell, it was not surprising to find that mtDNA encoded subunits of OXPHOS displayed the highest expression levels (**Figure 2A**). However, there was still some notable heterogeneity in expression. MT-ND4L, MT-ND6, and MT-ATP8 displayed some of the lowest levels of expression, which also corresponded to decreased numbers of cells expressing said transcripts (**Figure 2B**). nDNA encoded OXPHOS subunits and assembly factors also showed heterogeneous expression, both in the amount and cell type (**Figure 2C**). For example, *UQCRQ*, a gene that encodes a subunit of ubiquinol-cytochrome c reductase (complex III), displays low levels of expression in most cell types, except plasmacytoid dendritic cells and plasma cells. Other genes, such as *FOXRED1*, a chaperone protein required for the function of mitochondrial complex I, was expressed in very low amounts in all cell types studied. In general, similar statements regarding heterogeneity of nDNA genes can be made for the percentage of cells expressing said genes (**Figure 2D**). *FOXRED1*, for example, was not only expressed at low levels across all cell types, but also detected in only about 1-3% of cells. To illustrate this heterogeneity within a single OXPHOS complex, we focused on cytochrome c oxidase (COX, complex IV), the penultimate complex of the respiratory chain. After mapping COX subunit and assembly genes across our identified populations (**Figures S2, 2E**), mtDNA encoded subunits *MT-CO1*, *MT-CO2*, and *MT-CO3* showed high levels of expression across all cell types, comparable to our heatmap. The remaining nuclear encoded subunits showed varying degrees of expression in amount and the number of positive cells. Interestingly, *COX4I1*, a subunit 4 isoform, showed higher and more widespread expression compared to other nuclear encoded subunits. This was contrasted by the reduced expression of *COX4I2*, a second subunit 4 isoform that in coordination with *COX4I1* responds to reduced oxygen availability and modulates the electron transport chain (18). To validate the RNA expression heterogeneity for mitochondrial genes seen in our cohort, we utilized the Single Cell Portal at the Broad Institute (singlecell.broadinstitute.org) to examine the same COX subunit and assembly factor genes. The dataset comprised 2 PBMC samples from healthy adults. Consistent with our observations, mtDNA encoded genes displayed high levels of expression, while nDNA encoded genes showed heterogeneous expression across the different cell types (**Figure S3A**). *MT-CO1*, *COX5A*, and *COX4I1* showed patterns of expression that were similar to our own data (**Figures S3B, 2E**). While some of these expression discrepancies may be related to mitochondrial content, respiratory complex stoichiometry, or the timing of coordinated gene expression, our observations raise an interesting question of whether respiratory chains are assembled in distinctive ways in different cell types to match bioenergetic requirements and function. Indeed, mammalian cells maintain a specific oxidative OXPHOS capacity to satisfy their bioenergetic needs. These differences can be partially traced to differences in mitochondrial protein composition and supercomplex constituents and formation (19, 20). Our findings also have importance for deleterious variants in MtD and suggest that immune phenotypes may be heterogeneous based on the affected gene.

### 3.3 Depletion of select immune cell populations in MtD

**Figure 3:**
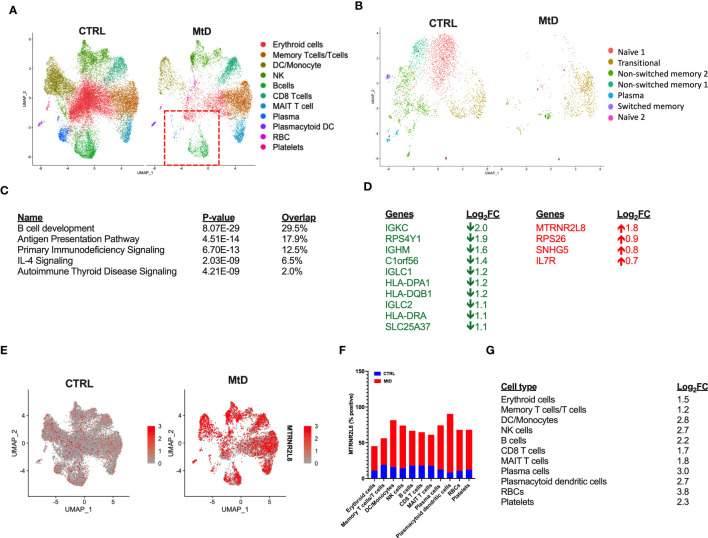
Differential gene expression. **(A)** Comparison of immune cell populations between children with MtD and controls *via* UMAP clustering. Red box = B cells and plasma cells. **(B)** B cell subpopulation analyses *via* UMAP clustering. **(C)** Top 5 gene ontology terms for overall differential gene expression profiles. **(D)** Table of Log_2_FC changes for differentially expressed genes (P < 0.05). Green = down, Red = UP. **(E)** Feature plot of *MTRNR2L8* expression across immune cell subsets. **(F)** Percent of cells that were positive for MTRNR2L8 within the identified clusters. **(G)** *MTRNR2L8* Log_2_FC within the individual immune subsets.

Based on the cellular identification, we next sought to compare the immune cell populations between our groups. Following normalization, several differences became apparent in children with MtD (**Figure 3A**). While platelets, plasmacytoid dendritic cells and various T cell populations (memory T cells/T cells, CD8 T cells, MAIT T cells) had cluster densities that were similar to controls, there was a marked depletion in remaining cell populations. Of note, select immune cell populations involved in antigen presentation and humoral immunity including B cells, plasma cells (**Figure 3A**, red box) and dendritic cells/monocytes were significantly affected.

To further refine our B cell populations, we performed a subcluster analysis, including the plasma cell cluster (**Figure 3B**). *Via* unsupervised clustering using a shared nearest neighbor approach, we segregated the subcluster into 7 populations (**Figure S4A**). Using established markers (**Figure S4B**) we were able to identify the following B cell subpopulations: transitional, naïve (2 populations), non-switched memory (2 populations), switched memory, and plasma cells. For nearly all B cell subpopulations, children with MtD displayed a prominent attenuation. Transitional B cells, however, seemed to fare better than other subtypes (Control = 403 cells, MtD = 512 cells). These cells serve as a provisional link between recent bone marrow emigrants and peripheral mature B cells (21).

Differential gene expression analysis can provide valuable insights into biological processes and potential molecular mechanisms underlying diverse phenotypes. To understand the marked differences in populations between the two groups, we first compared global gene expression profiles. Not surprisingly, given our UMAP clustering plots, the overall top canonical pathways included B cell development, antigen presentation, and primary immunodeficiency signaling (**Figure 3C**). The identification of these canonical pathways was also reflected in the downregulation of several genes involved in the B cell receptor (*IGKC, IGHM, IGLC1, IGLC2*) and antigen presentation (*HLA-DPA1, HLA-DQB1, HLA-DRA*) (**Figure 3D**). Interestingly, one of the most upregulated genes overall and within individual clusters was *MTRNR2L8*, a nuclear gene implicated as a marker of cell stress in multiple disease states (22). To visualize the expression of *MTRNR2L8* amongst different cell populations, we next projected this gene onto our UMAP clusters (**Figure 3E**). Compared to control subjects, *MTRNR2L8* showed enrichment in most clusters in children with MtD, despite overall reduced cluster densities. This pattern of *MTRNR2L8* expression also extended to our B cell subclusters (**Figure S4C**). Cell populations with the highest frequency of *MTRNR2L8* expression included plasmacytoid dendritic cells (90%), dendritic cells/monocytes (82%), plasma cells (74%), and NK cells (74%) (**Figure 3F**). Based on the widespread expression of *MTRNR2L8*, we next asked about the level of expression within different cell populations. *MTRNR2L8* expression was most elevated (log_2_FC > 2) in red blood cells > plasma cells > dendritic cells/monocytes > natural killer cells = plasmacytoid dendritic cells > B cells (**Figure 3G**). Notably, T cell populations were relatively lower for *MTRNR2L8* expression, when compared to other cell types. In general, we observed an inverse relationship between cluster densities and *MTRNR2L8* expression, suggesting that cell populations with higher expression were experiencing greater amounts of stress resulting in lower cell numbers. These results suggested that under certain conditions in MtD, select immune cell populations may also be affected under conditions of stress.

### 3.4 MtD lymphoblastoid cell lines express MTRNR2L8 and mir4485 under conditions of bioenergetic stress

**Figure 4:**
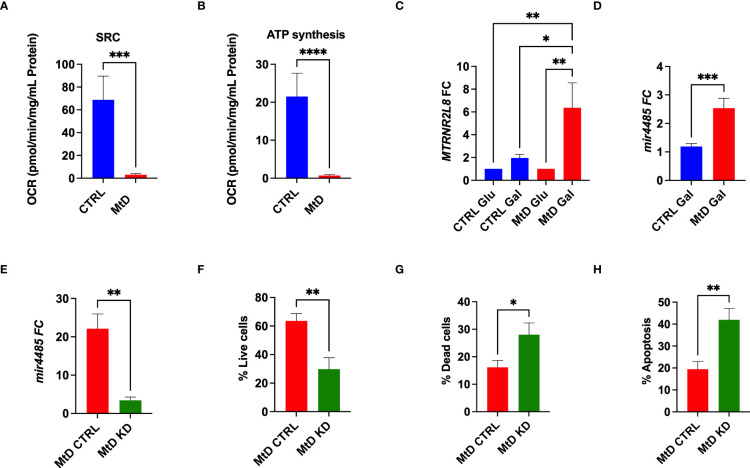
Lymphoblastoid cell lines express *MTRNR2L8* and *mir4485* under conditions of bioenergetic stress. **(A)** Spare respiratory capacity by extracellular flux analysis (N = 5/group). **(B)** ATP production by extracellular flux analysis (N = 5/group). **(C)** *MTRNR2L8* expression in lymphoblastoid cell lines cultured in galactose to promote OXPHOS. **(D)** *mir4485* expression under galactose conditions. **(E)** *mir4485* knockdown using antisense oligonucleotides. CTRL = transfection with scrambled antisense. **(F)** Staining for live cells by flow cytometry. **(G)** Staining for dead cells by flow cytometry. **(H)** Staining for apoptosis by flow cytometry. *P < 0.05, **P < 0.01, ***P < 0.001, ****P < 0.0001.

In order to corroborate the association of *MTRNR2L8* expression with a cellular stress phenotype in MtD, we studied lymphoblastoid cell lines (LCLs) from the same children with MtD. By extracellular flux analysis, we confirmed significant OXPHOS deficiency in the LCLs, as marked by depressed ATP synthesis and spare respiratory capacity (**Figures 4A, B**). Bioenergetic stress conditions were induced by incubating cells in galactose to facilitate the development of an oxidative metabotype (23). Under these experimental conditions, we observed ≈3-fold increase in *MTRNR2L8* expression over controls (**Figure 4C**). Contained within *MTRNR2L8* is *mir4485*, a miRNA that modulates mitochondrial complex I activity, ATP and ROS production, apoptosis, and glycolysis, and may be partially responsible for cytoprotective mechanisms (24, 25). *mir4485* was increased 2.5X over controls in LCLs from children with MtD.

Based on our results, we hypothesized that select cell populations were depleted due to their susceptibility to bioenergetic stress and that *mir4485* represented a cellular adaptation to foster survival in the remaining cells. Indeed, *mir4485* has been suggested to be involved in cellular stress responses and the regulation of apoptosis, but the response may be cell type dependent (24, 25). To define the function of this miRNA, we performed knockdown experiments with antisense oligonucleotides against *mir4485* in the same LCL cells. *mir4485* expression was decreased by 86% in our knockdown cells. Next, we examined cell survival. MtD LCLs with *mir4485* knockdown displayed a 52% decrease in the number of live cells (**Figure 4F**), with an increase in dead cells and cells undergoing apoptosis (**Figures 4G, H**). In summary, we propose that *mir4485* expression occurs under conditions of bioenergetic stress in MtD and promotes immune cell survival by modulating apoptosis. Therefore, in conjunction with our RNAseq studies, our results suggest that depleted cell populations (e.g., B cells and plasma cells) were bioenergetically stressed and lacked adequate reserves for survival, leading to apoptosis. Cells from depleted populations that were able to survive was due to their ability to upregulate *mir4485* expression. Our results also suggest that other cell types (e.g., T cells) may differ in their susceptibility to apoptosis due to bioenergetic stress as evidenced by their survival and lower expression of *MTRNR2L8*.

### 3.5 Children with MtD have an asynchronous relationship with viral infection

**Figure 5:**
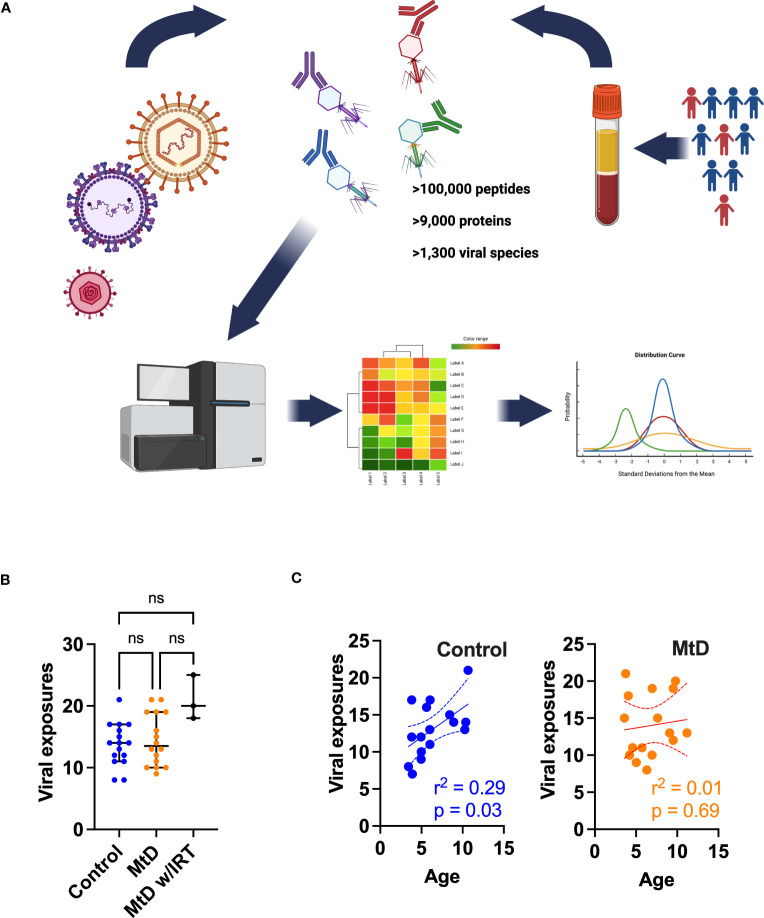
Viral exposures as determined by AVARDA. **(A)** Workflow for VirScan and AVARDA analyses for controls (N = 16) and MtD (N = 16). Serum was extracted from blood samples. Phage display library encoding the entire human viral proteome was constructed. Reactive phages were sequenced. Peptides were reassembled into viral proteins. AVARDA was used to deconvolute the antibody repertoire and make viral exposure calls. A viral exposure was called positive if the FDR < 0.05. **(B)** Viral exposures for individuals. Median and 95% confidence interval shown. MtD, mitochondrial disease; MtD w/IRT, mitochondrial disease on immunoglobulin replacement therapy. **(C)** Linear regression for viral exposures and age. Dashed lines indicate 95% confidence interval. ns, not significant.

To better understand the consequences of an apparent impairment in antigen presentation and humoral immunity, we wanted to characterize an integrated immune response that captured these critical aspects of immunity. Generally speaking, viral infections are inevitable, being more common amongst young children, due to incomplete immunity, and represent a communal challenge to the developing immune system (26). This shared experience of viral infection becomes an important tool for learning about humoral immunity under physiologic and pathologic conditions. Recently, a comprehensive and validated research platform called VirScan has been developed to query the human viral exposome (27). VirScan uses a phage display library containing a synthetic viral proteome to capture reactive antibodies. Next generation sequencing of phages provides a readout of viral peptides that can be assembled into viral proteins, indicating an individual’s viral exposome (**Figure 5A**). Using VirScan, we performed a cross-sectional study against the human viral proteome to identify antiviral IgG antibodies indicating previous exposure (N=16 controls, N=16 MtD, N = 3 MtD w/IRT [immunoglobulin replacement therapy], **Table S1**, subjects 1-19). The phage display library contained >100,000 peptides representing >9,000 proteins and >1,300 species. To account for cross-reactivity, we used the AntiViral Antibody Response Deconvolution Algorithm, known as AVARDA, to make calls on viral exposures (14). A viral exposure was considered positive with a conservative false discovery rate (FDR) < 0.05.

Prior to profiling the antiviral antibody repertoire *via* VirScan, we first wanted to address potential bias in our population that may shape humoral responses. Major Histocompatibility Complex (MHC) class II molecules (HLA II in humans), found on antigen-presenting cells, present processed antigens to CD4^+^ T cells. The activation of these T cells leads to the release of cytokines, which stimulate B cells to proliferate and differentiate into plasma cells and memory B cells, thereby driving the humoral immune response. To answer this question of bias, we performed HLA II typing in controls and MtD (**Table S2** and **Supplementary Excel file**) using oligonucleotide probes and flow cytometry. DNA samples were available for 13/16 controls and 18/19 children with MtD. Overall, the demographics did not differ between the 2 groups in regards to gender and race (p > 0.05). The predominant HLA II antigens detected were HLA-DRB1 and HLA-DQB1. The differences in these HLA II antigen distributions were also not statistically significant (p > 0.05). Our results indicated the absence of bias in HLA II based antigen presentation that could potentially skew the humoral response.

With these reassuring data, we next proceeded to profiling the antiviral antibody repertoire *via* VirScan. In our study, controls and children with MtD had similar profiles with a median of ≈14 viral exposures each (**Figure 5B**). As expected, MtD w/IRT had a higher number of exposures (median = 20) due to the pooled nature of human immunoglobulin therapy. Since children are exposed to an increasing number of viral infections as they age (28), we next asked whether viral exposures were related to age. Viral exposures exhibited a significant positive relationship with age in controls (r^2 =^ 0.29, P = 0.03), while children with MtD (r^2 =^ 0.01, P = 0.69) failed to show this relationship (**Figure 5C**).

### 3.6 The cadre of viruses is similar between MtD and controls

**Figure 6:**
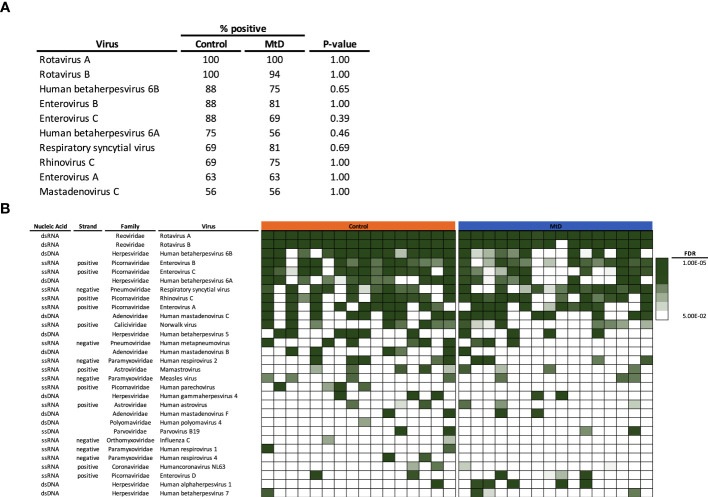
Viral exposome defined *via* AVARDA. Serum was analyzed from controls (N = 16) and MtD (N = 16). To account for overrepresentation and antigenic overlap, AVARDA was used to deconvolute the antibody repertoire and make viral exposure calls. By alignment of all library peptides to each other and all human viruses, AVARDA provides a probabilistic calculation of infection. A viral exposure was called positive if the FDR < 0.05. **(A)** Top 10 viral exposures. **(B)** Top 30 viral species are listed. Heat map of false discovery rates was constructed, in each row, each box represents an individual. MtD, mitochondrial disease; FDR, false discovery rate.

Given that the number of viral infections was similar between children with MtD and controls, we next asked whether the types of viral exposures were similar. *Via* AVARDA, we found that both controls and children with MtD demonstrated antibodies against comparable viral species (**Figures 6A, B**). We did not find enrichment of any particular virus in children with MtD (P > 0.39), such as norovirus, which can cause chronic infection in children with common variable immunodeficiency (29). Not surprisingly, the top 10 identified viruses comprised mostly respiratory and enteric infections, with Rotavirus A and B being the most common viruses encountered. The first decade of life represents a critical stage of development, as the adaptive immune system evolves due to microbial challenges and vaccination (28, 30). Accordingly, we conducted a sub analysis of this immune epoch in controls (N=13) and MtD (N=15). While the top ten viruses were similar to our entire cohort, interestingly, HSV-1 exposure was found in 5/15 MtD and in 0/13 controls (**Figure 6B**, P=0.04) in this age group. Recognition was supported by antibodies to multiple peptides in HSV-1 envelope glycoproteins D and G, and capsid scaffolding protein (**Figure S5**).

### 3.7 MtD show less robust antibody responses with limited diversity to shared viral proteins

**Figure 7:**
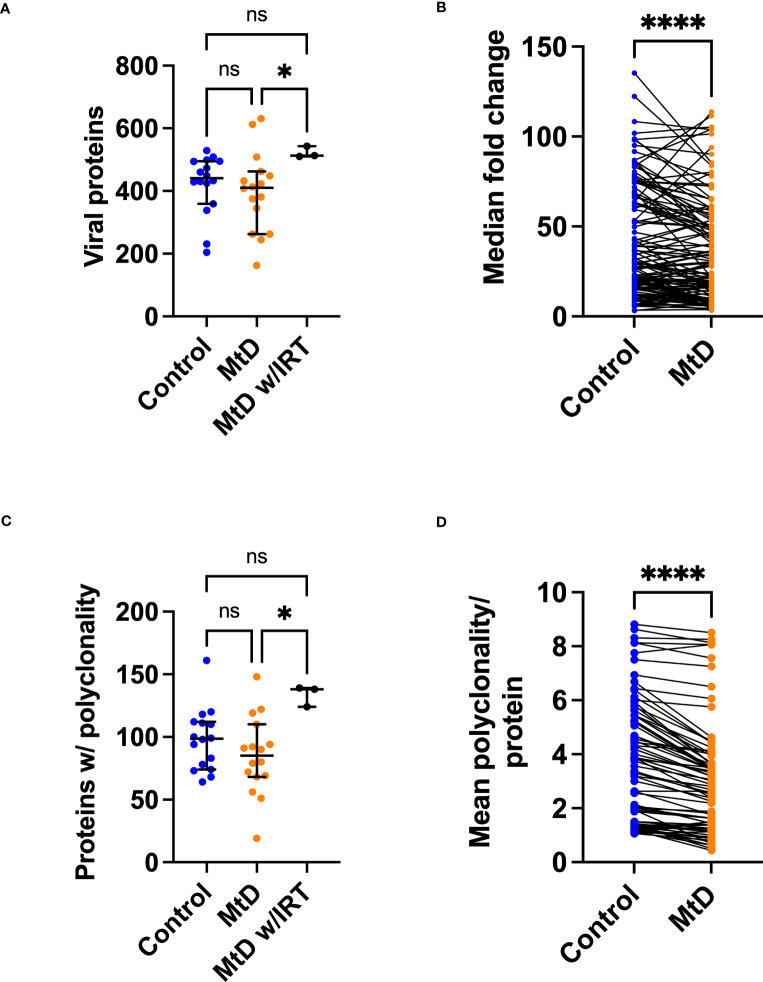
Viral proteins recognized by serum antibodies. Serum was analyzed from controls (N=16) and MtD (N=16). Children with MtD on IRT therapy (N=3) were analyzed as a separate group. **(A)** Total viral proteins per individual. Antibodies against viral proteins were called as a hit if the -Log P value ≥3. Median and 95% confidence interval shown. **(B)** Fold change of shared peptides. Using the shared 133 proteins identified, maximum protein fold changes were compared. **(C)** Total proteins with polyclonality per individual. A protein was marked as polyclonal if it was recognized by >1 antibody. Median and 95% confidence interval shown. **(D)** Polyclonality of shared peptides. Using the shared 54 proteins identified in E., the median polyclonality per protein was determined and the same proteins were compared between groups. ****P<0.0001 by Wilcoxon test. MtD = mitochondrial disease.

With the viral exposomes being highly similar, this presented an opportunity to examine the characteristics of the antiviral antibody repertoire which may impact overall immunity. To understand the breadth of antigens, we defined the total number of viral proteins recognized (**Figure 7A**). MtD recognized a median of 411 proteins (4.4% of library), while controls recognized a median of 441 proteins (4.7% of library). Total protein counts did not show any relationship with age for either group (**Figure S6A**). As expected, MtD on IRT (N=3) recognized a higher number of proteins with a median of 513 (5.4% of library), indicating a broader antibody repertoire.

Since individual total protein hits do not delineate common epitopes for comparisons, we next decided to define the number of shared viral proteins (**Figure 7B**). Considering the distribution of viral proteins, 133 were shared between controls and MtD. Next, by examining these shared proteins, we made comparisons of the magnitude of antibody responses. In general, higher levels of antibodies result in enhanced protection, with deficiencies representing a limitation in immunity (31). To answer this, we compared the 133 shared proteins expressed as fold change (FC) over background (**Figure 7B**). Although the antiviral antibodies were directed against proteins with similar functions (**Table S3**), we found lower fold changes in MtD indicating that their antibody responses to shared viral proteins were less robust.

Many viruses induce a strong polyclonal B cell response, ensuring that a single viral protein is recognized *via* overlapping epitopes by multiple B cell clones (32). Polyclonality exerts a protective effect by increasing the probability of recognizing an antigen and offers an initial defense against rapidly replicating microorganisms (33). To define polyclonality, we counted the number of proteins with >1 reactive antibodies (**Figure 7C**). In controls, a median of 99 proteins showed polyclonality. In MtD, that number was lower at a median of 85 proteins; however, this finding was not significant. Due to the diversity of the antibody repertoire, MtD w/IRT had higher degrees of polyclonality, as reflected by a median of 138 proteins. There was no relationship between polyclonality and age or number of viral exposures noted for either group (**Figures S6B, C**).

To compare polyclonality between groups, we next defined unique and shared proteins for comparison (**Figure 7D**). Regarding the distribution of proteins displaying polyclonality, 54 were shared. For shared proteins, we next asked whether polyclonality was similar. We found that MtD had lower amounts of polyclonality (P < 0.0001), signifying limitations in repertoire diversity.

## 4 Discussion

Over 50 mouse models for studying mitochondrial biology have been reported to date, several of which have been integral to studying the role of OXPHOS in immune cell function (5). Due to its phylogenic position amongst mammals, experimental utility and convenience, and the overabundance of immunologic and genetic tools and resources, the mouse is the principal mammalian model for studying human physiology and disease. Despite this established status, there are many recognized differences between mouse and human immunology (34). Therefore, including humans as the “highest order experimental system” (2) is an imperative for translational immunometabolism studies. To better understand the role of OXPHOS in humoral immunity, we performed single-cell RNAseq studies on children with MtD and matched controls. To help determine whether OXPHOS deficiency may produce an immune phenotype, we began by examining genes involved in MtD across various immunocyte subsets. We found that immunocyte populations showed differential expression of various OXPHOS components. Next, we turned our attention to comparing children with MtD with controls. In MtD, immunocytes involved in antigen presentation and humoral immunity showed signs of cellular stress resistance and associated depletion. To define the potential consequences of this pathology, we studied a shared immunologic experience amongst children (i.e., viral infection) and conducted a cross-sectional study of antiviral antibodies using PhIPseq (i.e., VirScan). While similar viral exposomes were found, we demonstrated that children with MtD have limitations in the antibody repertoire.

OXPHOS assembly requires the coordinated expression of genes encoded in two physically separate genomes. The mitochondrial genome encodes 13 subunits, while the nuclear genome encodes over 100 subunits and cofactors for OXPHOS assembly. Genes for OXPHOS complexes more often co-express amongst each other, rather than with genes of other complexes (35), suggesting a coordinated mechanism. This is akin to genes involved in metabolic pathways, which also show similar regulation (36). Despite this coordinated expression, OXPHOS genes display significant differences in relative mRNA abundance across different tissues, despite being housekeeping genes (36). Similarly, in our study, OXPHOS genes showed heterogeneity in expression across the various immunocytes studied. These differences may be related to the energy requirements of each cell type. It also implies that mitochondrial respiratory chains may be constructed differently in various immune cell types. Indeed, compared to bacteria, mitochondrial respiratory chain complexes in mammals contain a greater number of subunits (37). These additional proteins, acquired over evolution, function in OXPHOS assembly and stability rather than catalytic activity (38). Furthermore, children with MtD, characterized by deleterious variants in nDNA or mtDNA, retain some level of OXPHOS function despite having a null mutation. These data argue that less complex respiratory chains with different efficiencies may exist in select mammalian immunocytes. Furthermore, the expression of all known genes for OXPHOS assembly and function may not be required. These assertions also have implications for mitochondrial medicine, where deleterious variants in genes involved in mitochondrial function may selectively affect immune cell subsets due to varying compositions or requirements of their respiratory chains.

During our characterization of immune cell subsets, *MTRNR2L8*, a marker of cellular stress resistance (39–41), was found to be highly expressed in PBMCs from children with MtD as well as LCLs from children with MtD following upregulation of OXPHOS. These results suggest that the induction of this stress marker is directly related to OXPHOS function. Interestingly, this marker also displayed greater expression in immune cell populations (e.g., monocytes/dendritic cells, NK cells, B cells, and plasma cells) that were also depleted in children with MtD, consistent with its role as a stress marker. This cellular stress was reproduced in lymphoblastoid cell lines when OXPHOS was required for the generation of ATP. T cells, in general, showed comparatively milder expression of *MTRNR2L8*, and a lack of T cell depletion in children with MtD. Our findings contrasted with those reported in a recent paper that demonstrated selection against OXPHOS deficiency in T cells specifically, with sparing of other immunocytes (42). These discrepancies may be related to the type of mutation studied and the difference in ages between the two cohorts. However, both sets of results suggest that immune cell populations may be differentially affected, depending on type of deleterious variant.

As a stress gene, *MTRNR2L8* has intriguing features that relate to its origins and mechanisms of action. Regarding its origins, *MTRNR2L8* descended from the mitochondrial 16S rRNA gene *MTRNR2. MTRNR2* also encodes humanin, a small biologically active peptide that can be translated in the mitochondria (21 amino acids) and the cytoplasm (24 amino acids) (22). *MTRNR2* was transferred to the nucleus during evolution and exists as multiple isoforms, including *MTRNR2L8. MTRNR2L8* is located on chromosome 11p15.4 and was initially considered to be a pseudogene. However, recent studies suggest that the MTRNR2-like series of genes may actually produce functional peptides like humanin-like protein 8. Furthermore, humanin-like protein 8 is similar in sequence to humanin and even contains a polymorphism that makes it identical (22). In general, humanins function intracellularly and extracellularly under conditions of cellular stress. Intracellularly, humanin may bind to BAX, sequestering it from the mitochondrial membrane, thus preventing apoptosis (43). Extracellularly, following binding to the CNTFR/WSX-1/GP130 receptor complex, humanin can induce neuroprotection against mechanisms of Alzheimer’s disease pathology in a neuronal cell line *via* a STAT3 related mechanism (44, 45). Concerning MtD specifically, elevated levels of humanin have been detected in the skeletal muscle of CPEO and MELAS patients (46, 47), and have been found to restore ATP levels in MELAS lymphocytes (48). *MTRNR2L8* also has an additional mechanism of action besides potentially encoding humanin-like protein 8. Buried within its intron is *miR-4485*, a micro-RNA that modulates complex I activity, ATP production, ROS production, caspase-3/7 activation, and apoptosis (24, 25). In addition to *MTRNR2L8*, bioenergetic stress in our LCL cells also induced the expression of *miR-4485*. Knockdown studies confirmed that *mir4485* functions in modulating cell death *via* apoptosis. Overall, the dual functions of *MTRNR2L8* and *mir4485* make it an interesting target for future studies on its role in MtD.

Amongst the most significantly affected populations (i.e., population density and *MTRNR2L8* expression) in our scRNAseq studies, were constituents of the humoral immune response, specifically B cells and plasma cells. During B cell activation and differentiation, multiple cellular processes ensure that B cells recognize a diverse array of microbial antigens (i.e., >10^12)^, undergo exponential expansion, and differentiate into effector cells (49). These characteristics of the polyclonal B cell response increase the probability of recognizing an antigen (33) and exerts a protective effect. Perturbations in antibody responses in children with MtD have been reported previously by our group; specifically, deficits in antibodies for vaccine preventable illnesses and the need for immunoglobulin replacement therapy (8, 50). In our current study, further characterization of the humoral immune response *via* the viral exposome showed that children with MtD have less robust antibody responses and less repertoire diversity. Indeed, robust and diverse antibody responses offer better protection against invading microorganisms (31). Although the reasons for these perturbations may be related to B cell generation or the germinal center response, our results suggest problems with maintenance of long-lived immunity. To date, the majority of the biomedical literature on mitochondria and the humoral immune response comes from mouse studies. Therefore, we will review our results in this context.

In peripheral lymphoid organs such as the lymph node or spleen, activated B cells form germinal centers where B cells exponentially expand, and the B cell receptor (BCR) undergoes somatic hypermutation to improve antigen affinity and class switch recombination to improve functionality. Activated and germinal center (GC) murine B cells markedly increase glucose uptake, glycolysis, mitochondrial mass and membrane potential, and the expression of tricarboxylic acid cycle and OXPHOS genes, developing a highly energetic and balanced (i.e., glycolysis + OXPHOS) metabotype (51–53). The absence of glucose, suppression of OXPHOS by oligomycin (52), or targeted disruption of cytochrome c oxidase (54), leads to a reduction in the number of GC B cells as well as affinity maturation and class switch recombination.

In addition to murine memory B cells, mitochondria are also critical for long-lived plasma cell survival (LLPCs). LLPCs reside in the bone marrow and are the primary producers of circulating antibodies, maintaining protective immunity against a multitude of viral pathogens. In fact, the bone marrow is the primary site of this long lived aspect of humoral immunity (55). Therefore, in the absence of infection VirScan measures LLPC output. Long-term maintenance of LLPCs is supported by CD28-mediated activation of NFkB and downstream IRF4. IRF4 not only promotes gene transcription programs for plasma cell development, but also upregulates glycolysis and OXPHOS and helps maintain mitochondrial homeostasis *via* biogenesis (56–58). The upregulation of glycolysis results in the complete oxidation of glucose, as disruption of pyruvate import by UK5099 decreases mitochondrial spare respiratory capacity (58). Therefore, children with MtD may have an inability to maintain long-lived plasma cells due to OXPHOS limitations in LLPCs, leading to perturbations in the antibody repertoire.

While our results have broad implications for the role of OXPHOS in humoral immunity, they also have important implications for children with MtD. Viral infections in children with MtD can cause metabolic decompensation, a potentially life-threatening scenario that can involve acute disease progression and worsening disability (7, 59). Which viral infections may cause metabolic decompensation remains a major unanswered question in mitochondrial medicine, and there are currently no formal efforts to understand the viral exposome in this vulnerable population. Similar to controls, children with MtD experience about 14 viral exposures with respiratory and GI viruses predominating. This number of viral exposures was slightly higher than what has been reported in adults and may represent waning immunity in adults, as well as more recent exposures in children (27). While infection in children is inevitable, our study demonstrated that the association between age and viral exposures is absent in children with mitochondrial disease. This asynchronous relationship with infection may be accounted for by risk mitigation behaviors, non-pharmaceutical interventions that help prevent viral exposure and illness (60).

In summary, our data demonstrate the effects of OXPHOS deficiency in select immune cell populations in humans, particularly components of humoral immune response. We also identified *MTRNR2L8* and *mir4485* in MtD, biomarkers of cellular stress, which are directly related to OXPHOS dysfunction, and are associated with perturbations in immunocyte populations. Our studies of the viral exposome provide an important translational link, particularly with regards to B cell maturation and long-term immune memory. Based on our findings, we propose that an integrated approach, studying models and patients with MtD, is a fundamental modus operandi for translational immunometabolism aimed at understanding the role of OXPHOS.

## Data availability statement

The scRNAseq data presented in the study are deposited in the GEO repository, accession number GSE234505.

## Ethics statement

The studies involving human participants were reviewed and approved by NIH Institutional Review Board. Written informed consent to participate in this study was provided by the participants’ legal guardian/next of kin.

## Author contributions

EG-L: study design, subject evaluation, acquiring data, writing. PB: data analysis, writing. JM: conducting experiments, acquiring data, data analysis. TT: conducting experiments, acquiring data, data analysis. SK: study design, subject recruitment, subject consent, acquiring data, writing. ET: subject recruitment, subject consent, acquiring data. DG, SZ and TW: data analysis, writing. NISC: acquiring data, analysis. WF: acquiring data, analysis, writing. PM: conceptualization, study design, acquiring data, data analysis, writing. All authors contributed to the article and approved the submitted version.
