# Supplementary material for: Primary oxidative phosphorylation defects lead to perturbations in the human B cell repertoire
Source: Front Immunol. 2023 Jul 7;14:1142634. doi: 10.3389/fimmu.2023.1142634 (PMC10361569; doi:10.3389/fimmu.2023.1142634)
Supplement: Supplementary file 1 [file DataSheet_1.pdf]

SUPPLEMENTARY DATA

Subject	Diagnosis	Age	Gene	CD3 #/uL	CD19 #/uL	CD16/56 #/uL	Ig levels (mg/dL)
1	LS	7.0	MT-ATP6	2844	459	239	850
2	MD NOS	3.6	EARS2	943	216	156	653
3	LS	3.7	MT-ATP6	1495	409	104	730
4	LS	4.1	SURF1	1258	370	310	586
5	LS	4.2	MT-ATP6	3073	688	480	859
6	LLS	4.4	NUBPL	1250	128	77	787
7	LS	4.6	MT-ND3<br>MT-ND2	2774	1306	643	848
8	LS	5.0	SERAC1	1160	367	403	824
9	LS	5.7	MT-ND1	2333	406	314	663
10	MD NOS	6.3	EARS2	1028	204	174	854
11	LS	7.0	C12ORF65	NA	NA	NA	NA
12	LLS	9.5	SDHA	1704	663	213	1016
13	LLS	7.6	NUBPL	1717	193	327	718
14	LS	9.1	MT-ND3	1687	224	222	906
15	LLS	9.8	MICU1<br>MT-CYB	NA	NA	NA	NA
16	MD NOS	8.0	MT-TL1<br>MT-TS	1291	440	228	903
17	KSS	9.5		1522	396	87	1091
18	LS	11.2	NDUFS2	2002	339	150	819
19	MD NOS	4.5	MT-CYB	1996	409	398	1083
20	LS	6.6	HIBCH	2253	592	139	746

Table S1: Clinical characteristics of subjects with MtD.
Clinical and molecular diagnostic characteristics of children with MtD. MtD = mitochondrial disease, LS = Leigh Syndrome, LLS = Leigh-like Syndrome, MELAS = Mitochondrial encephalopathy lactic acidosis and stroke, KSS = Kearns Sayre Syndrome, MtD NOS = Mitochondrial disease not otherwise specified, CD3 = T cells, CD19 = B cells, CD16/56 = NK cells. Green = < 10th %ile for age, Red = >10th %ile for age.

	Human subjects			
	Patients	Controls	Total	p value
Male/female ratio				
Male	9	8	17	0.7168
Female	9	5	14	
Subjects (total)	18	13	31	
Race				
White	11	6	17	0.6061
Black/African American	2	3	5	
Other	5	4	9	
Subjects (total)	18	13	31	
HLA-DRB1				
DRB1*01	2	1	3	0.6187
DRB1*03	4	1	5	
DRB1*04	7	5	12	
DRB1*07	7	5	12	
DRB1*08	2	1	3	
DRB1*11	3	5	8	
DRB1*15	6	1	7	
DRB1*16	2	2	4	
Other antigens	3	5	8	
Antigens (total)	36	26	62	
HLA-DQB1				
DQB1*02	8	4	12	0.9226
DQB1*03	14	11	25	
DQB1*04	4	2	6	
DQB1*05	4	3	7	
DQB1*06	6	6	12	
Antigens (total)	26	36	62	

Table S2: HLA II typing. DNA samples from controls (N=13) and children with mitochondrial disease (N = 18) were used for HLA typing for HLA class II loci (HLA-DRB1, -DQB1, -DRB3, -DRB4, -DRB5) with sequence specific oligonucleotide (SSO) probes and flow cytometry. Fisher's exact test (male/female ratio), Chi square test 5x5 (Race and DQB1) and unlimited contingency table (DRB1), two-sided with $p < 0.05$ considered statistically significant.

GO ID	Function	Control (%)	MtD (%)
GO:0019062	virion attachment to host cell	118 (5)	95 (5)
GO:0003723	RNA binding	89 (4)	73 (4)
GO:0006351	transcription, DNA-templated	85 (3)	72 (3)
GO:0003968	RNA-directed 5'-3' RNA polymerase activity	84 (3)	70 (3)
GO:0005524	ATP binding	80 (3)	67 (3)

Table S3: Viral protein functions. GO = gene ontology.

A

Sample	% Viability
CTRL1	97.9
CTRL2	98.4
CTRL3	99.0
CTRL4	96.5
MtD1	99.4
MtD2	98
MtD3	99.7
MtD4	97.6

B

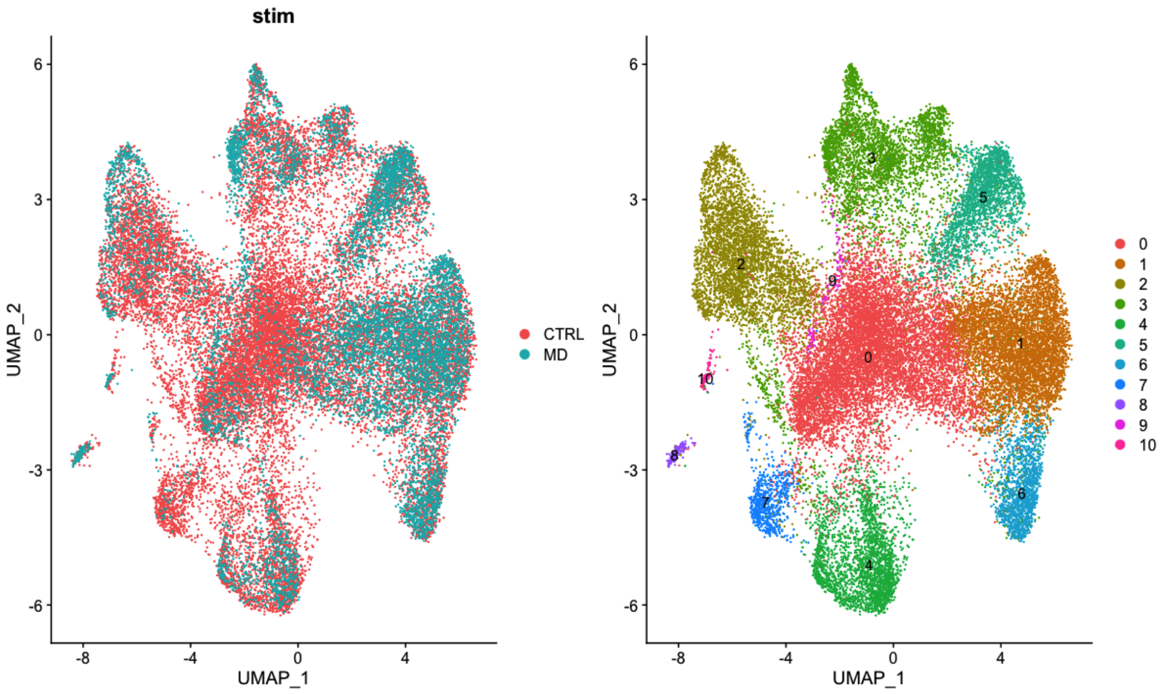

Figure S1: Definition of immune cell clusters. Children with MtD (N = 4) and Controls (N =4) were combined to determine markers for immune cell populations. A) Cell viability for the samples studied. B) Overlay of peripheral blood mononuclear cells from control and MtD subjects (left). Separation of immune cell clusters by unsupervised clustering via Uniform Manifold Approximation and Projection (right).

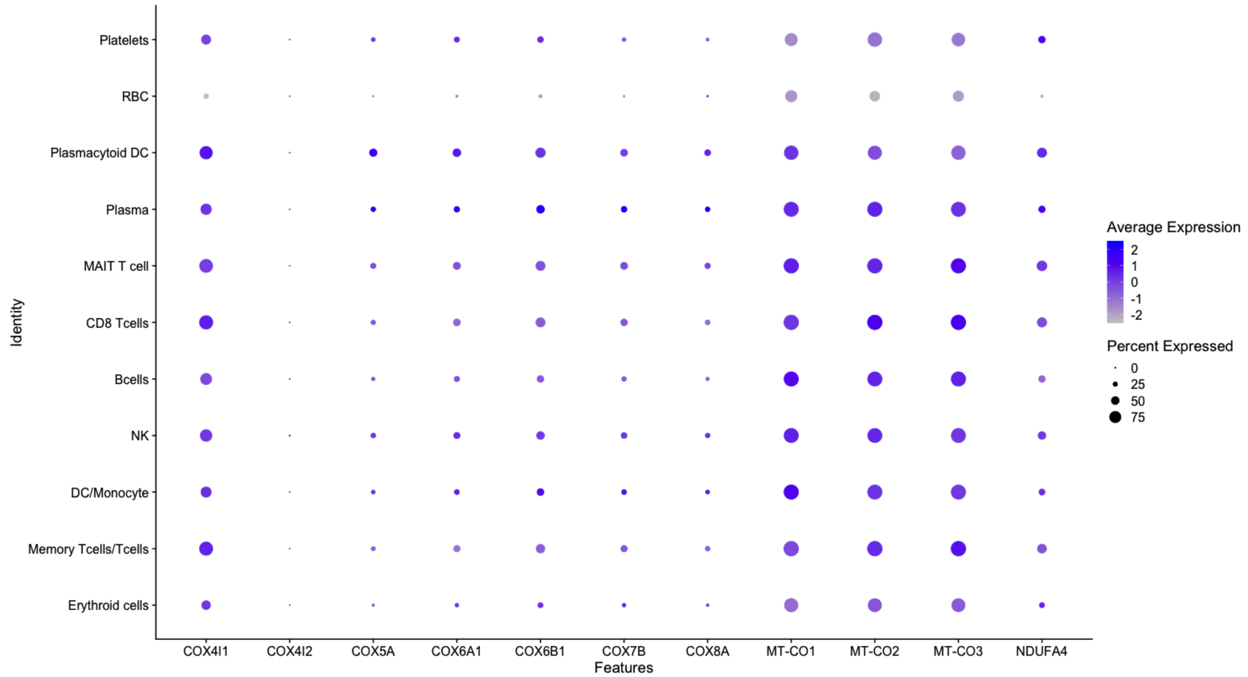

Figure S2: Dot plot for nDNA and mtDNA encoded subunits of cytochrome c oxidase. Combined immune cell subsets were studied for their expression of cytochrome c oxidase subunits.

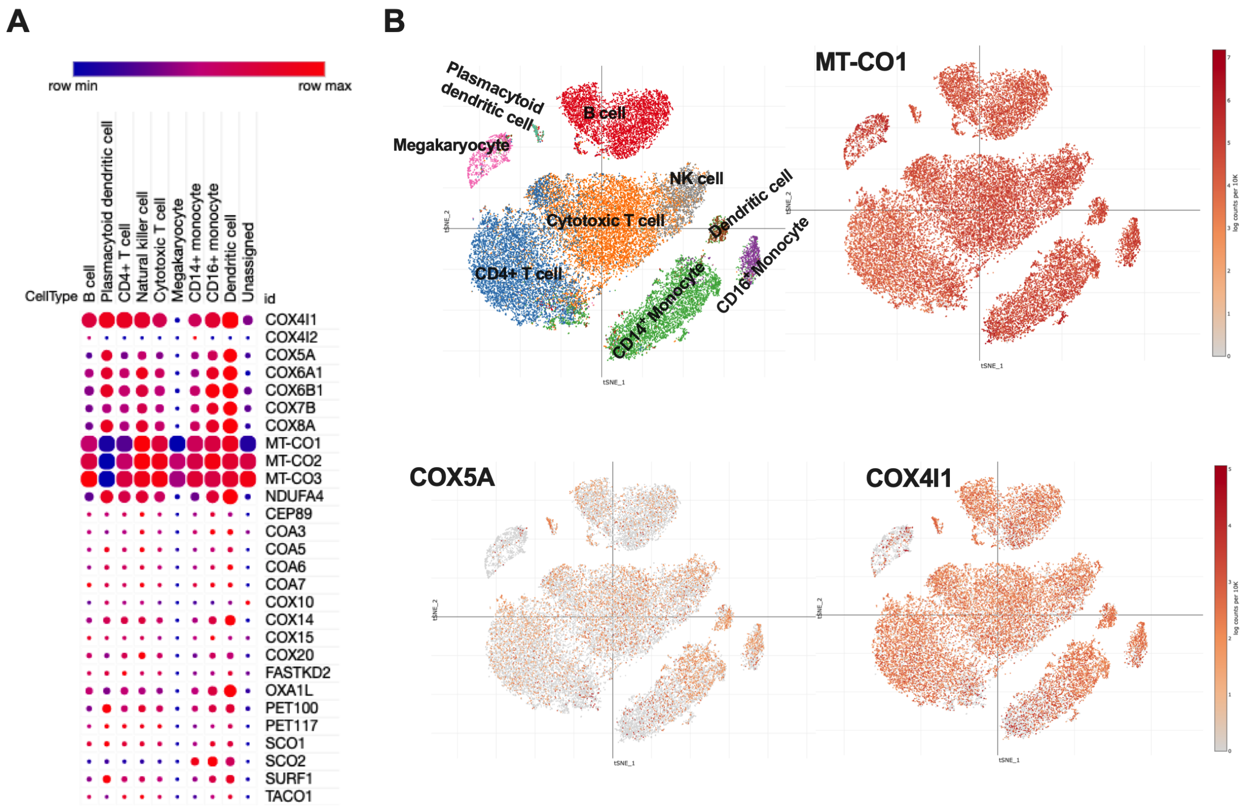

Figure S3: Validation dataset for expression of cytochrome c oxidase subunits. Data was abstracted and analyzed from the Single Cell Portal of the Broad Institute (singlecell.broadinstitute.org). Data is representative of two peripheral blood mononuclear cell samples from healthy volunteers. A) Comparison of gene expression for nDNA and mtDNA encoded genes involved in cytochrome c oxidase function. B) Comparison of select cytochrome c oxidase genes projected onto feature plots of immune cell subsets.

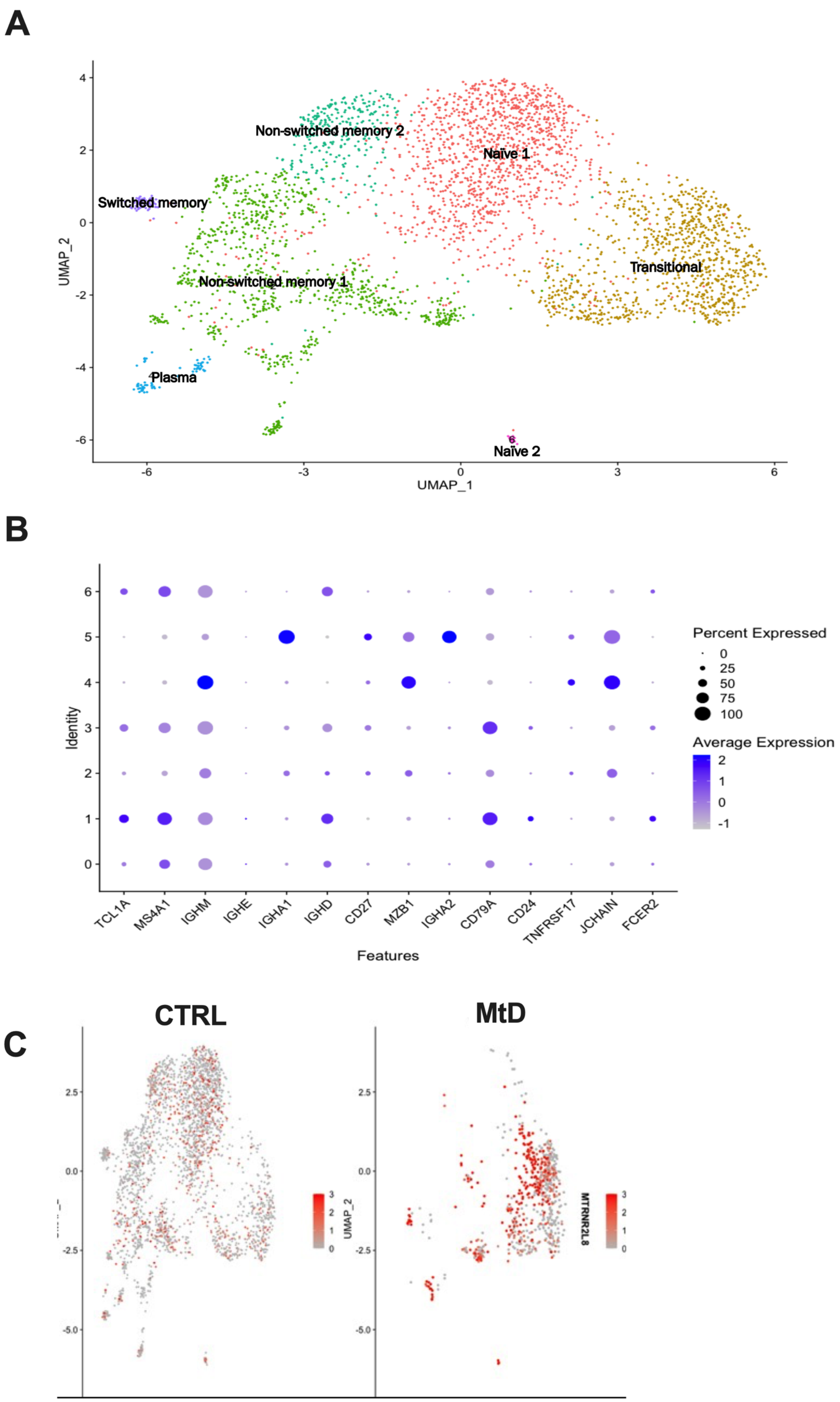

A
Non-switched memory 2
Naïve 1
Switched memory
Transitional
Non-switched memory 1
Plasma
Naïve 2
UMAP_1
UMAP_2
B
Percent Expressed
0
25
50
75
100
Average Expression
2
1
0
-1
Identity
TCL1A
MS4A1
IGHM
IGHE
IGHA1
IGHD
CD27
MZB1
IGHA2
CD79A
CD24
TNFRSF17
JCHAIN
FCER2
Features
C
CTRL
MtD
UMAP_2
MTRNR2L8

Figure S4: B cell subcluster determination. Children with MtD (N = 4) and Controls (N =4) were combined to determine markers for B cell populations. A) Separation of B cell clusters by unsupervised clustering via Uniform Manifold Approximation and Projection. B) Differential gene expression analyses identified cell specific markers. A dot plot for cellular marks and their alignment with various cell populations was constructed. C) Feature plot of *MTRNR2L8* expression across B cell subsets.

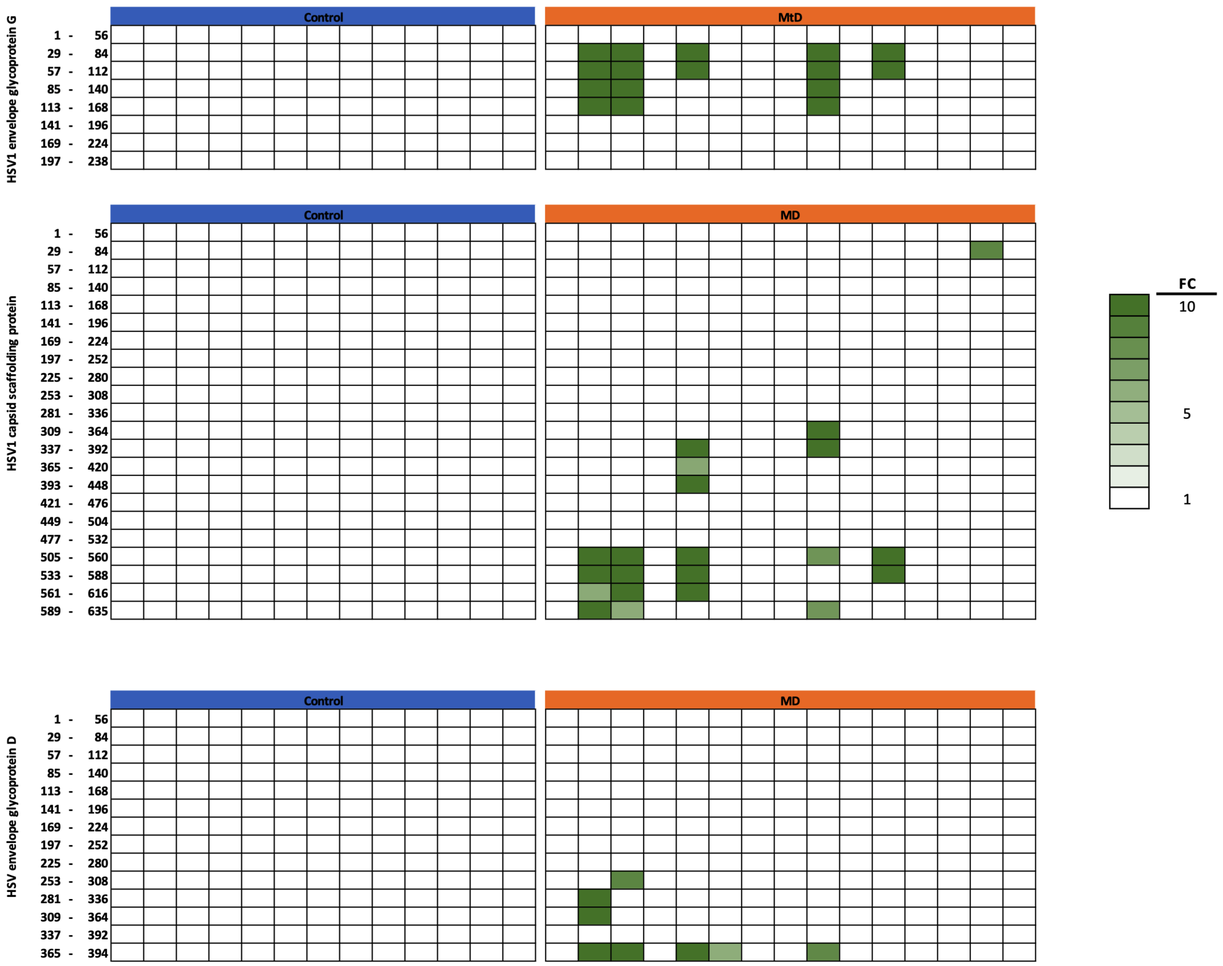

Figure S5: HSV-1 epitopes in controls and children with MtD. Controls (N=13) and children with MtD (N=15) who are in the first decade of life were studied for antibodies against HSV-1 epitopes. Numbers in left hand column indicate amino acid positions. FC = Log_2 fold change.

A

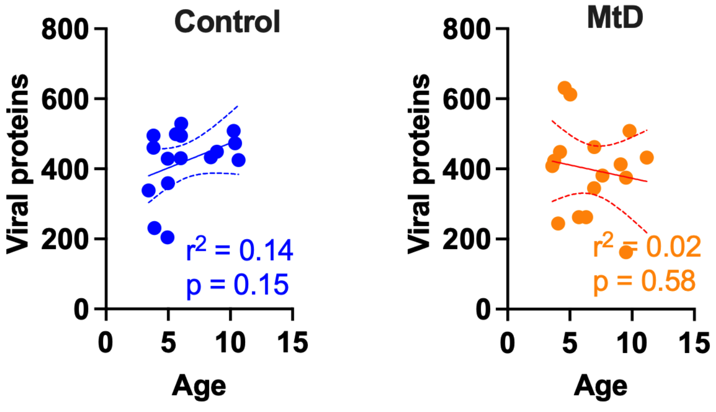
Control
Viral proteins
800
600
400
200
0
0
5
10
15
Age
r^2 = 0.14
p = 0.15
MtD
Viral proteins
800
600
400
200
0
0
5
10
15
Age
r^2 = 0.02
p = 0.58

B

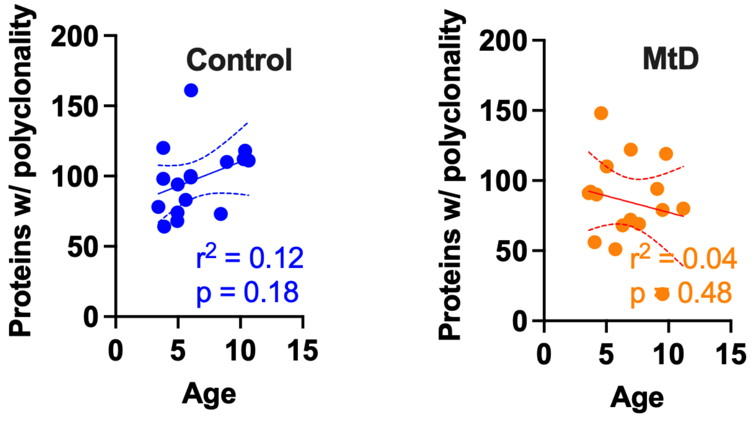
Control
Proteins w/ polyclonality
200
150
100
50
0
0
5
10
15
Age
r^2 = 0.12
p = 0.18
MtD
Proteins w/ polyclonality
200
150
100
50
0
0
5
10
15
Age
r^2 = 0.04
p = 0.48

C

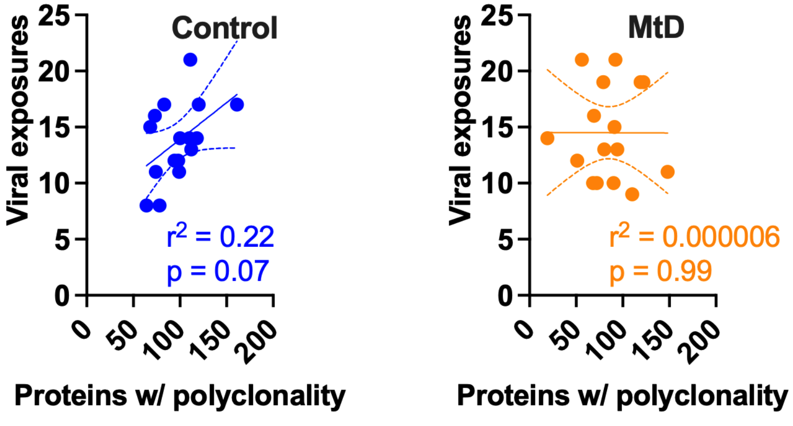
Control
Viral exposures
25
20
15
10
5
0
0
50
100
150
200
Proteins w/ polyclonality
r^2 = 0.22
p = 0.07
MtD
Viral exposures
25
20
15
10
5
0
0
50
100
150
200
Proteins w/ polyclonality
r^2 = 0.000006
p = 0.99

Figure S6: Regression analyses for number of viral proteins, viral exposures, age and polyclonality. Control (N = 16) and children with mitochondrial disease (N = 16) were anlyzed by linear regression to describe contributions of independent variables. A) The effect of age on the number of viral proteins recognized. B) The effect of age on polyclonality. C) The effect of polyclonality on the number of viral exposures.
